# The in vitro micronucleus assay using imaging flow cytometry and deep learning

**DOI:** 10.1038/s41540-021-00179-5

**Published:** 2021-05-18

**Authors:** Matthew A. Rodrigues, Christine E. Probst, Artiom Zayats, Bryan Davidson, Michael Riedel, Yang Li, Vidya Venkatachalam

**Affiliations:** Amnis Flow Cytometry, Luminex Corporation, Seattle, WA USA

**Keywords:** Cell biology, Biomarkers

## Abstract

The in vitro micronucleus (MN) assay is a well-established assay for quantification of DNA damage, and is required by regulatory bodies worldwide to screen chemicals for genetic toxicity. The MN assay is performed in two variations: scoring MN in cytokinesis-blocked binucleated cells or directly in unblocked mononucleated cells. Several methods have been developed to score the MN assay, including manual and automated microscopy, and conventional flow cytometry, each with advantages and limitations. Previously, we applied imaging flow cytometry (IFC) using the ImageStream^®^ to develop a rapid and automated MN assay based on high throughput image capture and feature-based image analysis in the IDEAS^®^ software. However, the analysis strategy required rigorous optimization across chemicals and cell lines. To overcome the complexity and rigidity of feature-based image analysis, in this study we used the Amnis^®^ AI software to develop a deep-learning method based on convolutional neural networks to score IFC data in both the cytokinesis-blocked and unblocked versions of the MN assay. We show that the use of the Amnis AI software to score imagery acquired using the ImageStream^®^ compares well to manual microscopy and outperforms IDEAS^®^ feature-based analysis, facilitating full automation of the MN assay.

## Introduction

The in vitro micronucleus (MN) assay is used worldwide to assess the ability of various chemicals or other agents to induce DNA damage in the fields of population biomonitoring and radiation biodosimetry^1–3^. The assay is also fundamental in genetic toxicology to test the development of chemicals, pharmaceuticals, and cosmetics for human use^4,5^. MN originate from whole chromosomes or chromosome fragments that fail to be incorporated into the main nucleus following nuclear division^5^ and consequently, MN frequency can be used as an endpoint to quantify DNA damage.

Scoring of MN is typically performed in once-divided binucleated (BN) cells by blocking division with Cytochalasin-B (Cyt-B). As the post-cellular division fate of MN is generally not well understood, the use of Cyt-B ensures that MN can be associated to a single cell division, circumventing confounding factors such altered division kinetics or cell cycle delays^5–8^. The Cyt-B approach also allows the scoring of mononucleated (MONO) and polynucleated (POLY) cells permitting the evaluation of cytotoxicity^9^. The assay can also be performed in the absence of Cyt-B, making it faster and easier to score, however it has been demonstrated that chemicals that weakly induce MN by strongly impacting cytostasis may produce false negatives^10,11^. The non-Cyt-B version of the assay is also limited by the formation of BN cells at low frequency, which may lead to erroneous results if unscored^7^. Despite these cautions, a number of publications have demonstrated that the non-Cyt-B version of the assay can detect significant increases in MN frequency in several cell lines using a variety of test chemicals^12–14^.

The MN assay has historically been scored using manual slide microscopy, which benefits from high-resolution imaging of nuclear and cytoplasmic cellular components, but is prone to scorer fatigue and variability^15^. In addition, prolonged use of a microscope can lead to repetitive stress injuries, including back, neck, and vision problems^16–18^. To address these difficulties, automated methods have been developed, including slide-scanning microscopy and flow cytometry. However, commercial automated slide-microscopy systems require high-quality slides with optimal cell density, which can be challenging to create at low cell concentrations, and lack cytoplasmic visualization, decreasing scoring robustness for MONO and POLY cells^19–23^. Conventional flow cytometry methods offer dramatically higher throughput compared to manual and automated microscopy methods. However, flow cytometric assays require a cell lysing step making it impossible to perform the Cyt-B version of the assay, and false positives may occur due to the formation of apoptotic bodies and other DNA positive debris^24,25^. Moreover, as a non-imaging method, flow cytometry may not be used to visually validate the legitimacy of purported MN populations.

In light of these limitations, we and others have reported on the use of imaging flow cytometry (IFC) to automate the MN assay for genetic toxicology^26–29^ and biodosimetry applications^30–35^, demonstrating that MN can be accurately scored at increased sample throughput without the requirement to prepare high-quality slides. The ImageStream^®X^ Mark II (Amnis^®^ Flow Cytometry, Luminex, Seattle, WA, USA) combines the speed and statistical robustness of conventional flow cytometry with high-resolution imagery capabilities of microscopy in a single system^36^. IFC technology has been used to quantify autophagy^37^ and extracellular vesicles^38^, to demonstrate diagnostic potential in acute leukemia^39^, for assessment of phagocytosis and NETosis^40^ and to quantify DNA damage via the chromosomal aberration assay^41^. The ImageStream^®X^ Mark II is accompanied by a powerful image analysis software package (IDEAS^®^) which permits robust analysis strategies to be created. However, the feature-driven nature of IDEAS^®^ requires expert knowledge and rigorous optimization to create an analysis strategy that correctly identifies subtle morphologies in some imagery (e.g. MN). Therefore, a more robust and readily accessible image-driven solution is desirable.

In this study, we developed a classification model using convolutional neural networks (CNNs) to score the MN assay, overcoming the limitations of feature-based image analysis. The use of deep learning for image analysis has grown significantly in recent years as it mimics the ability of the human brain to discern patterns^42^. In particular, CNNs which are multi-layered neural networks that use kernel-based processing, are particularly effective at extracting information at multiple levels to comprehensively characterize image data, thereby facilitating enhanced discrimination of populations or identification of image subtleties^43^. As such, CNNs have been used in a number of fields to enhance accuracy and improve workflow including pathology^43^, cell biology^44^, and pharmaceuticals^45^. One disadvantage of CNNs has been the requirement for computer scientists to perform optimization and validation, making them relatively inaccessible to researchers lacking expertise in those areas. To address this limitation, Amnis^®^ AI (AAI) software has been developed to allow researchers to directly develop, train, and validate CNN models for IFC data through the use of a convenient graphical user interface. A second disadvantage of CNNs is their requirement for large training datasets in order to achieve high accuracy. Consequently, IFC data are particularly well suited for classification by CNNs as large numbers of images can be automatically acquired at rapid collection speeds. The development of CNN-based classifiers using AAI is further supported by unique clustering and prediction functionalities which permit rapid ground truth model class assignment of morphologically similar images, greatly streamlining the construction of large training datasets. In a recent publication, we demonstrated that a model created and trained with the AAI software using only Brightfield imagery was able to robustly identify and differentiate silicone oil droplets and protein aggregates, a distinction of particular importance in the development of therapeutic protein formulations^45^.

With respect to the MN assay, image classification by CNNs present several advantages in comparison to traditional feature-based scoring methods, including the elimination of complex image analysis strategies, translatability across multiple cell lines and test chemicals, and higher resistance to experimental perturbations. In this study, the AAI software was used to develop a single classification model to score both the cytokinesis blocked and unblocked versions of the MN assay using TK6 lymphoblastoid cells and three widely used test chemicals; Mitomycin C (MMC), Etoposide, and Mannitol. Scoring was performed using manual microscopy, IDEAS^®^ and AAI to identify all key events^15^ to assess genotoxicity and cytotoxicity. Our results show that AAI outperforms the feature-based IDEAS^®^ analysis and compares well to manual slide microscopy.

## Results

### Deep-learning model development using Amnis^®^ AI

**Fig. 1:**
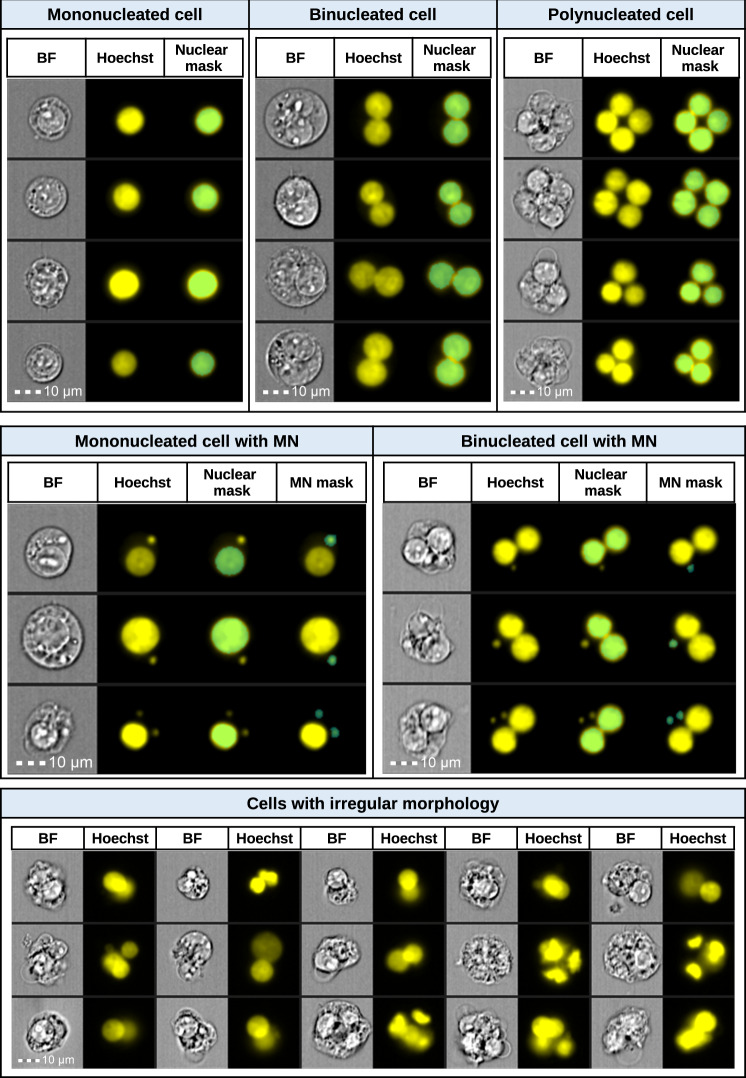
Representative IFC imagery of all key events that must be scored in the in vitro micronucleus (MN) assay. Key events include mononucleated cells (with and without MN), binucleated cells (with and without MN), and polynucleated cells. Cytoplasmic material is visible in the Brightfield imagery and DNA content (e.g. main nuclei and MN) is visualized in the Hoechst (yellow) channel. Masks (cyan) overlaid onto the images identify either the main nuclei or the MN. Representative imagery of events with irregular nuclear morphology that do not fit the published scoring criteria are also shown.

To quantify genotoxicity and cytotoxicity by microscopy methods, individual cellular images must first be categorized by number of nuclei and MN^4^. In the Cyt-B version of the assay, the critical events that must be scored are MONO cells, BN cells with and without MN, and POLY cells^9^. In the non-Cyt-B version of the MN assay, the critical events that must be scored are MONO cells with and without MN^9^, but it has also been demonstrated that scoring BN cells with MN can yield valuable information^7,8^. Example images selected from IFC data show that cytoplasm and DNA content (e.g. nuclei and MN) can be visualized within all key events (Fig. 1). Additionally, as with microscopy, some imagery captured using IFC will possess irregular morphology as defined by the published scoring criteria^15^ and must be excluded from scoring, including images with irregularly shaped and/or overlapping nuclei, nuclei within the same cell that differ from one another in size and/or staining intensity and imagery where individual nuclei are not clearly identifiable. In IDEAS^®^, all nuclei and MN can be automatically identified using masks that identify and highlight pixels within a defined region of interest in an image (Fig. 1). Image-based features can then be used to create a gating strategy that permits critical events that meet the published scoring criteria to be quantified while concurrently removing all other events. The details of the IDEAS analysis strategy used in this work can be found in previous publications^26,27^.

**Fig. 2:**
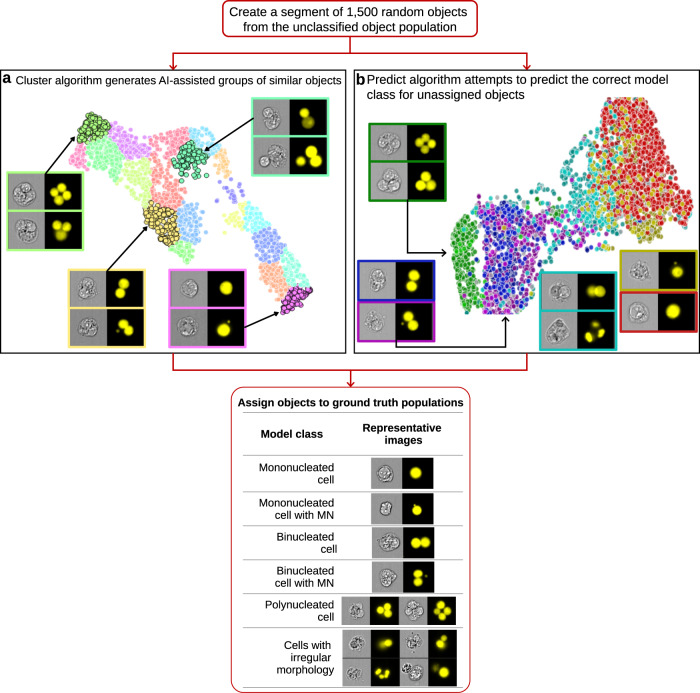
Diagrammatic view of the Amnis® AI (AAI) software workflow. The user loads IFC data files into the software using a step-by-step wizard. The Segment option is then used to randomly select 1500 objects, at which point either the Cluster or Predict algorithm can be selected. The Cluster algorithm (**a**) is used to group like objects within a segment together based on the imagery of both the unclassified and the ground truth objects. However, at this stage, the untrained model has difficulty differentiating between images with subtle morphological differences such as BN cells with and without MN (yellow cluster), hence these images will be placed into the same cluster. The Predict algorithm (**b**) requires a minimum of 25 images in each of the ground truth model classes and attempts to predict the correct model classes for all unclassified objects within a segment. The predictions made by the Predict algorithm on the unclassified objects are based on the morphology of the objects that have been assigned to the specific ground truth model classes. The predict algorithm is better able to identify subtle morphological differences between images, such as BN cells without MN (blue) versus BN cells with MN (purple). Objects such as these will appear in close proximity on the object map, but the user can examine the images in each predicted class separately. Using the results of both the Cluster and Predict algorithms, the user can then manually assign objects (individually or in large groups) to their appropriate ground truth model classes. Ground truth model classes were populated with objects for all critical events in the MN assay (i.e. MONO cell, MONO cell with MN, BN cell, BN cell with MN, POLY cells and cells with irregular morphology). The model was then constructed by splitting the ground truth imagery into training, validation, and test sets using an 80/10/10 ratio. Following training and testing, additional datasets were classified using the model.

The AAI software was used to train a model that sorted images into six classes using both the BrightField (BF) and nuclear images: MONO cell, MONO cell with MN, BN cell, BN cell with MN, POLY cells, and cells with irregular morphology. The workflow used to build and train a model in the AAI software is shown in Fig. 2. MMC data consisting of a total of 300,144 images from both Cyt-B (15 data files) and non-Cyt-B (10 data files) experiments across the dose range were loaded into the AAI software. Each data file contained initial ground truth populations consisting of 50 images (where possible) per model class that had been hand-tagged in IDEAS^®^. Once the data and initial ground truth populations were loaded into the AAI software, the segment option was used to create a segment of 1500 randomly selected objects. Segments of 1500 objects are used in the AAI software to improve the speed with which the Cluster and Predict algorithms run, both of which can be executed once a segment has been created (Fig. 2). Objects can then be assigned to the ground truth model classes using either the Cluster or Predict algorithms.

**Fig. 3:**
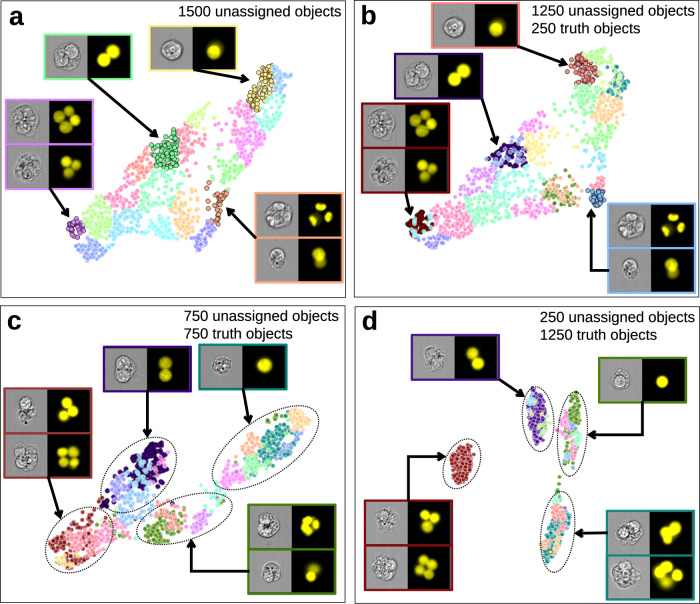
Examples of how the cluster algorithm attempts to group similar objects within a segment. Beginning with 1500 unassigned objects (**a**), the Cluster algorithm groups objects of similar morphologies into clusters of similar objects located close to one another on the object map. By assigning 250 (**b**), 750 (**c**), and 1250 (**d**) objects to the appropriate ground truth model classes, the accuracy with which the unassigned objects are grouped by the algorithm increases. In panels (**c**) and (**d**), distinct islands have become more visible and it can be seen that MONO, BN and POLY cells are well-separated from one another, while objects with irregular morphology have also been placed into their own discrete island.

The Cluster algorithm attempts to group morphologically similar objects within a segment. First, each image passes through a pre-trained CNN. The features computed at the final layer of the CNN just prior to classification are used as inputs to a dimensionality reduction algorithm to aid in visualization. Ground truth is not required, but if labels are provided they will be leveraged to improve the results of the dimensionality reduction. Five times more clusters than classes are assigned to ensure that the clusters contain substantially similar images. The result presents the user with a list of clusters that can be selected to view the objects within a particular cluster in the image gallery. Additionally, an interactive object map is displayed, where clusters can also be selected to view the associated imagery (Fig. 2). Individual objects, groups of objects within a cluster, or entire clusters can then be assigned to the appropriate ground truth model class. Initially, since the model has not been trained, images with MN will be clustered together with images that do not have MN as the morphological difference between the two is very subtle. Figure 2a shows that while the cluster algorithm can differentiate between MONO (purple cluster), BN (yellow cluster), POLY (green cluster) and irregular (cyan cluster) classes, differentiating between images with and without MN at this stage is difficult. Figure 3 demonstrates how the cluster algorithm groups similar objects using only the MONO, BN, POLY and irregular classes as an example. Figure 3a shows the initial clustering step with 1500 unassigned objects being grouped into clusters of objects with similar morphology, where MONO and POLY cells are clustered toward opposite sides of the object map as their morphologies are very different. BN cells are clustered towards the middle of the object map, while objects with irregular morphology are dispersed throughout the central portion of the object map as these objects are more morphologically heterogeneous when compared to all other model classes. As more objects are assigned to ground truth model classes (Fig. 3b-d), clusters become increasingly separated on the object map, indicating the AAI model is learning which particular images belong to the classes of interest. In Fig. 3d, four distinct islands are clearly visible, with each island representing a single class. Once all classes have been populated with at least 25 ground truth objects, the Predict algorithm (Fig. 2b) can be used to assign unclassified objects to the class of best fit. The predict algorithm trains a simple linear model using the features extracted from the pre-trained CNN. The predicted classes created match the classes in the ground truth data and objects that do not fit well into any class are labeled as unknown. In this work, Fig. 2b demonstrates that the predict algorithm creates six model classes and plots them on the object map: MONO (red), MONO with MN (yellow), BN (blue), BN with MN (purple), POLY (green) and irregular (cyan). The morphological differences between the MONO and POLY class are readily identified, hence their positioning at opposite ends of the object map permitting rapid addition into the ground truth data. Conversely, for example, the morphological differences between BN cells with and without MN are very subtle and as such, the two populations are essentially mixed on the object map. However, the user can view all imagery in the predicted classes for both populations which makes rapid population of the ground truth datasets achievable because objects are pre-sorted into the highest probability class. This functionality is especially useful for identifying rare objects which are laborious to identify in a non-sorted data set, such as low-frequency MONO and BN cells with MN.

Iterating between the Cluster and Predict algorithms, 190 segments containing 285,000 objects were examined. Objects were assigned to the appropriate ground truth model classes until all classes contained between 1500 and 10,000 images. A total of 31,500 objects, 10.5% of the total number of objects loaded, were used to train the model. Model training was completed within the AAI software when the accuracy on the training and validation datasets converged and reached a maximum which required 70 epochs. Accuracy statistics including precision, recall and F1 score are available metrics in the AAI software for evaluating the efficacy of a trained model. These are common metrics used in machine learning, where precision is the percentage of events that were correctly classified, recall is the percentage of truth events that were correctly classified and the F1 score conveys the balance between precision and recall. In all cases, the higher the value is, the more accurate the model is. For each model class, the values for these statistics ranged from 86.0% to 99.4% with an overall weighted average model accuracies of 96.0%, 96.3%, and 95.6% for the training, validation, and testing datasets, respectively (Table 1).

**Table 1:** True positive (TP), false positive (FP) and false negative (FN) numbers as well as accuracy statistics for the training, validation and testing datasets use in the AAI MN model.

	Training data						Validation data						Testing data					
Model class	TP	FP	FN	Precision (%)	Recall (%)	F1 (%)	TP	FP	FN	Precision (%)	Recall (%)	F1 (%)	TP	FP	FN	Precision (%)	Recall (%)	F1 (%)
Mononucleated cell	3561	149	39	96.0	98.9	97.4	982	21	18	97.9	98.2	98.1	972	19	28	98.1	97.2	97.6
Mononucleated cell with MN	1189	43	11	96.5	99.1	97.8	148	11	2	93.1	98.7	95.8	146	16	4	90.1	97.3	93.6
Binucleated cell	3522	97	78	97.3	97.8	97.6	986	20	14	98.0	98.6	98.3	978	21	22	97.9	97.8	97.8
Binucleated cell with MN	1193	77	7	93.9	99.4	96.6	147	12	3	92.5	98.0	95.1	147	16	3	90.2	98.0	93.9
Polynucleated cell	2724	115	76	95.9	97.3	96.6	341	19	9	94.7	97.4	96.1	333	15	17	95.7	95.1	95.4
Cells with irregular morphology	3175	155	425	95.3	88.2	91.6	430	33	70	92.9	86.0	89.3	435	52	65	89.3	87.0	88.1
Overall weighted average					96.0						96.3						96.6	

### Microscopy, IDEAS^®^, and AAI dose-response for Cyt-B version of the assay

**Fig. 4:**
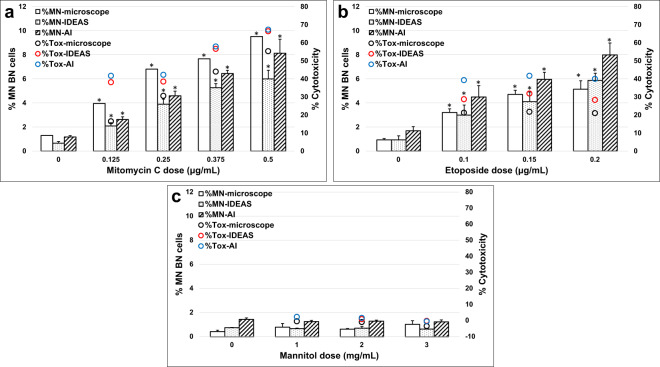
Genotoxicity measured in the Cyt-B version of the MN assay. The percentage of MNed BN cells by microscopy (clear bars), IDEAS (dotted bars), and Amnis AI (striped bars), as well as cytotoxicity measured by microscopy (black circles), IDEAS (red circles), and Amnis AI (blue circles) are shown following a 3 h exposure and 24 h recovery for: (**a**) Mitomycin C (MMC), (**b**) Etoposide and (**c**) Mannitol. Statistically significant increases in MN frequency compared to solvent controls are indicated by asterisks (*P ≤ 0.001).

Dose-response data following exposure to MMC, Etoposide, and Mannitol for the Cyt-B version of the assay are shown in Fig. 4 and Table S1. Across all experiments and scoring methods, the mean background MN frequencies for all solvent controls ranged from 0.43 to 1.69% which compares well to recently published data demonstrating that MN frequencies from negative control using TK6 cells ranged from 0.32 to 1.38% when scored by either microscopy or flow cytometry^46^. For both MMC and Etoposide, statistically significant increases (*p* < 0.001) in the number of MNed BN cells were observed in all doses tested when compared with solvent controls. For MMC and Etoposide, the MN frequencies in the dosed samples ranged from 2.09 to 9.50% and 2.99 to 7.98%, respectively across all scoring methods and AAI scoring compared well to microscopy. For Mannitol no statistically significant increases in MN frequency were observed at any dose, as expected, and the MN frequencies across all samples ranged from 0.43 to 1.45%. For IDEAS^®^ and AAI scoring, cytotoxicity values were higher overall in all MMC and Etoposide dosed samples when compared to microscope scoring but showed similar trends across the dose range (Fig. 4). Only the top MMC dose exceeded 55 ± 5% cytotoxicity as recommended by the OECD Test Guideline 487^4^. For Mannitol, cytotoxicity values were similar for all scoring methods, ranging from −3.5 to 2.2% across the dose range.

On average, the MMC samples required 1–2 min to collect 15,000 images in each data file, and the Etoposide and Mannitol samples required 5–6 min to collect 30,000 images per data file. The AAI software data processing time was less than one minute per file and an overall average of 6493 MONO cells, 7733 BN cells and 2649 POLY cells were scored by the AAI model. In all cultures but one (one replicate of 0.5 µg/mL MMC), over 2000 BN cells were scored in all data files, double the requirement specified in the OECD guideline^4^.

### Microscopy, IDEAS^®^, and AAI dose-response for non-Cyt-B version of the assay

**Fig. 5:**
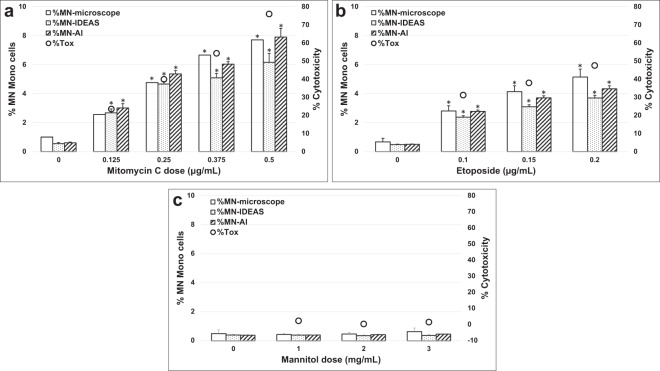
Genotoxicity measured in the non-Cyt-B version of the MN assay. The percentage of MNed MONO cells by microscopy (clear bars), IDEAS (dotted bars) and Amnis AI (striped bars) and cytotoxicity determined by cell counts (black circles) are shown following a 3 hr exposure and 24 hr recovery for: (**a**) Mitomycin C (MMC), (**b**) Etoposide and (**c**) Mannitol. Statistically significant increases in MN frequency compared to solvent controls are indicated by asterisks (*P ≤ 0.001).

**Fig. 6:**
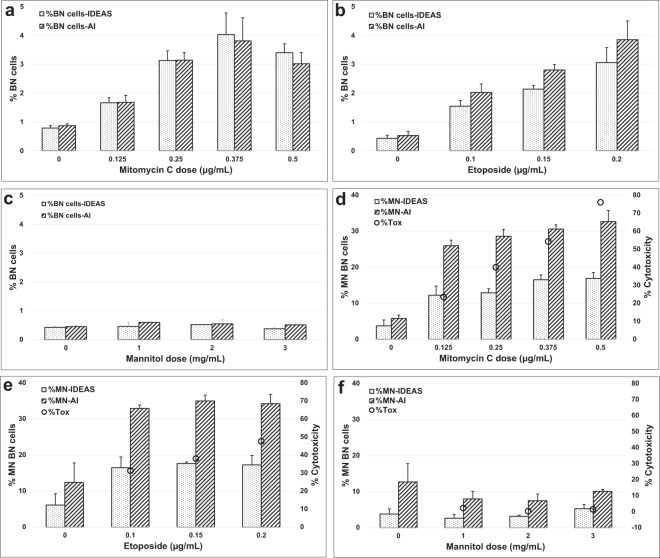
Frequency of BN cells and MNed BN cells in the non-Cyt-B version of the MN assay. The percentage of BN cells quantified by IDEAS (dotted bars) and Amnis AI (striped bars) for (**a**) Mitomycin C (MMC), (**b**) Etoposide and (**c**) Mannitol. The percentage of MNed BN cells by IDEAS (dotted bars) and Amnis AI (striped bars) are shown for (**d**) MMC, (**e**) Etoposide and (**f**) Mannitol and following a 3 hr exposure and 24 h recovery. Cytotoxicity quantified by cell counts is also indicated (black circles).

TK6 dose response to MMC, Etoposide and Mannitol for the non-Cyt-B version of the assay are shown in Fig. 5 for MNed MONO cells, Fig. 6 for MNed BN cells and Table S2. Across all experiments and scoring methods, the mean background MN frequencies in MONO cells for all solvent controls ranged from 0.38 to 1.0% which compares well to historical results^46^. For both MMC and Etoposide, statistically significant increases (*p* < 0.001) in the number of MNed MONO cells were observed when compared with solvent controls for all methods and doses except the 0.125 µg/mL MMC dose point using microscopy. For MMC and Etoposide, the MN frequencies in the dosed samples ranged from 2.55 to 7.89% and 2.37 to 5.13%, respectively across all scoring methods and again, AAI scoring compared well to microscopy. For Mannitol no statistically significant increases in MN frequency were observed at any dose, as expected, and the MN frequencies across all samples ranged from 0.36 to 0.63%. Similar to the Cyt-B experiments, only the top MMC dose exceeded 55 ± 5% cytotoxicity^4^ (Fig. 5). For Mannitol, cytotoxicity values were also similar to the Cyt-B experiments and ranged from 0.2 to 2.2% across the dose range. On average, the MMC samples required 30 s to collect 10,000 images in each data file, and the Etoposide and Mannitol samples required 2 min to collect 30,000 images per data file. The AAI software data file processing time was less than one minute per file. For the MMC samples processed using AAI, an overall average of 8121 MONO cells were scored and for the Etoposide and Mannitol samples, an overall average of 27,866 MONO cells were scored. This data demonstrates that many more cells can be acquired and scored per culture in just a few minutes by IFC when compared to microscopy, in which 1,000 MONO cells per culture are typically scored^4^.

Previous studies have demonstrated that genotoxins can cause cell cycle delays, resulting in an increase in BN cells of which a substantial proportion contain MNl; ranges of 9.4–40.4% have been reported^7^. It has been suggested that not scoring these cells and only scoring MNed MONO cells may lead to false negative results when using weak genotoxins^7,8^. However, due to the rarity of spontaneous BN cells combined with the low throughput of slide-based microscope scoring, this is a tedious task. Fortunately, this becomes straightforward using IFC and AAI given the large number of images that can be acquired and automatically scored. Figure 6a-c and Table S2 show that overall, the rate of BN cells increased with increasing dose due to reduction in cell proliferation following exposure to both MMC and Etoposide. For MMC, Etoposide and Mannitol the rate of BN cells identified by AAI reached a maximum of 3.80%, 3.06%, and 0.602% respectively. Within these BN cells, the average MN frequencies scored by AAI for MMC, Etoposide and Mannitol were 29.4%, 34.0%, and 5.5%, respectively (Fig. 6d–f).

## Discussion

This paper presents the use of a deep learning software package to automate analysis of the MN assay. Recent publications have demonstrated that easy-to-use, interactive tools permit researchers to use pre-defined deep learning models^47^, or train new models^48^ to analyze their image data without the requirement for computational expertise. The AAI software utilized in this paper is entirely user interface-based and has been architected to work with large datasets. To address the requirement for large training datasets using CNN-based classifiers, AAI is equipped with unique Cluster and Predict functionalities that allow rapid assignment of images to ground truth model classes (Figs. 2 and 3). Previously, we demonstrated that the IFC-based MN assay offers simple sample preparation and staining procedures along with straightforward data acquisition. Additionally, we have shown that the IDEAS^®^-based data analysis strategy quantifies all key events in the MN assay, though the image analysis is complex and in some cases required optimization when translating across cell lines and chemicals^26,27^. The results presented in this paper demonstrate how a deep learning model trained in AAI software is able to robustly identify cell morphologies of interest yielding results comparable to visual microscopy, while eliminating the requirement for image analysis expertise and optimization required by the IDEAS^®^-based approach.

In this study, MMC, Etoposide and Mannitol were evaluated for genotoxicity and cytotoxicity using microscopy, IDEAS^®^ scoring and AAI scoring. In both Cyt-B and non-Cyt-B versions of the assay, increases in cytotoxicity were observed with increasing dose when compared to solvent controls using all scoring methods for both MMC and Etoposide as expected with a 3 h exposure + 24 h recovery schedule^8,12,13^. While MMC cytotoxicity increased with dose, with Etoposide in the Cyt-B version of the assay, all doses produced similar levels of cytotoxicity, which was also observed by Elhajouji using a similar exposure/recovery schedule^12^. With Mannitol, no significant increases in cytotoxicity were observed using any scoring method in either version of the assay, which is consistent with results from previous work by our group and by others^26,49^. When comparing cytotoxicity values obtained by the three scoring methods, AAI gave slightly higher values, which may be attributed to differences in manual scoring with microscopy and lower scoring of polynucleated cells by IDEAS (Fig. 4).

When evaluating genotoxicity through MN quantification, clear positive responses (*p* < 0.001) when compared with solvent controls were observed for MMC and Etoposide at all doses for IDEAS^®^ and AAI scoring in both the Cyt-B and non-Cyt-B versions of the assay (Figs. 4, 5). With microscope scoring, statistically significant MN increases were not observed for the lowest doses of MMC and Etoposide in the Cyt-B version of the assay and for the lowest dose of MMC in the non-Cyt-B version of the assay. No statistically significant increases in MN frequency were observed using Mannitol for any scoring method. Overall, MN frequencies compared well across all three scoring methods for both versions of the assay but in general, AAI scoring compared better to microscopy than did IDEAS^®^ scoring, which may be attributed to several factors. In previous work^26^ it was demonstrated that an analysis strategy in IDEAS^®^ could be used to automate scoring of the assay. However, due to the rigidity of the masking parameters in IDEAS^®^, some smaller MN may be missed when using compounds that are weak inducers of MN or when MN arise from chromosome fragments breaks and are small. This work demonstrates that AAI can more robustly identify more subtle morphologies, such as smaller MN, MN that reside very close to the nuclei and MN that are slightly out of focus in the IFC imagery. Moreover, by combining IFC data with AAI scoring, several thousand MNed BN and MONO cells can be scored, providing a more robust quantification of genotoxicity compared to slide-based microscopy.

In recent publications, it has been suggested that certain genotoxins can cause cell cycle delays which result in MNed BN cells accumulating in culture. Sobol et al. reported on the implications of cell cycle delays impacting MN frequencies following exposure of TK6 cells to three well-known genotoxins, including MMC and Etoposide, and suggested an extended recovery time may be necessary to accurately quantify MN frequencies^8^. Additionally, Doherty *et al*. quantified MNed BN cells in the non-Cyt-B version of the assay using TK6 cells after showing that not scoring these cells could lead false negative results^7^. Following a 24 hr exposure, mean BN cell and MN BN cell rates in dosed samples reached as high as 40.4%; however, the number of MNed BN cells scored was less than 50 from most samples^7^. In this work, we have shown that MNed BN cells can be scored automatically in the non-Cyt-B version of the assay. While the average rate of BN cells scored by AAI was lower in dosed samples when compared to the results shown by Doherty et al. due using only a 3 h exposure, an average of 194 MNed BN cells were scored across all dosed samples. These results confirm findings in previous publications but also demonstrate the potential to shorten protocol schedules since many more cells can be scored using IFC, which may provide faster time-to-result. Additionally, this solution is more elegant as it can replace tedious microscope scoring and the complexities of feature-based image analysis since a single AI model permits scoring of all key events in both versions of the assay.

In a previous publication, we demonstrated that when using the IDEAS^®^ analysis strategy on IFC data captured at 60× magnification, the overall average MN frequency was below the historical range^26,46^. In this work, the mean MNed BN cell and MNed MONO cells compare well to historical data and can likely be explained by the extended depth of focus at lower magnifications and the use of the AAI software. The 40× magnification used in this work provides a 4 µm depth of focus compared to a 2.5 µm depth of focus available at 60× magnification used previously^26^. Therefore, some legitimate MN that reside at a slightly different depth of focus than the main nuclei will appear brighter at 40× and can be scored more robustly. Use of the 40× magnification objective, combined with the use of AAI permits correct identification of some legitimate but dimmer MN that may be missed with the 60× objective and IDEAS^®^ scoring.

With the introduction of the AAI software to replace feature-based image analysis to score the IFC-based in vitro MN assay, the advantages over microscopy and conventional flow cytometry are further realized. In previous work, we discussed the superior throughput and automation of IFC in comparison to other techniques used to perform the MN assay. With IFC, several thousand images can be collected and analyzed in just a few minutes, and the use of the 96-well plate autosampler permits unattended acquisition of image data. We have observed a 5-fold reduction in the batch processing time required when using AAI to process data in comparison to IDEAS^®^ due to the fact that once CNN models have been trained, they can rapidly be applied to classify new data. One of the most significant advances that this work demonstrates is the use of a single deep learning model to classify new data in both the Cyt-B and non-Cyt-B versions of the MN assay. This offers a significant advancement over microscopy, especially with the Cyt-B version of the assay that requires two scans (one for genotoxicity and a second for cytotoxicity assessment) and over IDEAS^®^ scoring where separate analysis templates are required for each version of the assay.

In a number of recent publications, several methods of image-based cell sorting have been described. The ability to sort and further analyze individual cells often overcomes the loss of information that occurs when attempting to extract subtle cell-to-cell variations from bulk populations^50^. With the advancements in computing power over the last decade, the ability to capture imagery and apply deep learning networks in near-real time to physically sort cells of interest based on morphological features is now possible. Several such systems have been described, employing various methods of image capture including frequency-division-multiplexed microscopy^51^, photomultiplier tubes^52^, interdigital transducers^53^, Raman scattering^54^, and virtual-freezing fluorescence imaging^55,56^. While each of these methods demonstrates significant advances to sort cells based on fluorescence and morphological differences, applying these systems to sort all key events in the MN assay may be challenging due to the very subtle morphology of the MN and the high resolution, focused imagery required for precise detection. However, as computing power improves and deep learning models become more advanced it may be possible to identify MN and other markers through a combination of morphology and fluorescence. For example, a multiplexed MN assay could be developed in which fluorescence is used to identify early and late apoptotic and necrotic cells that could be automatically quantified and sorted. Additionally, since MN form their own nuclear envelope, a marker that attaches to the nuclear lamina could be incorporated into sample preparation protocols and a CNN-based model could be trained to identify this characteristic during image acquisition. Furthermore, the addition of pan-centromeric probes or anti-kinetochore antibodies may allow for the differentiation of MN that contain centromere positive chromosomes from those that are composed of only acentric chromosome fragments^57^. This may allow for the development of an AI-assisted imaging flow cytometry workflow to sort cells containing MN that have been generated from aneugenic versus clastogenic compounds. These cells could then be further analyzed to fully investigate additional markers that may require higher resolution imagery (e.g. 100X) to visualize such as telomere markers^58^, a process that would be made more efficient if microscope slides contained only micronucleated cells.

This paper demonstrates the use of a deep learning model constructed within the AAI software package to perform the in vitro MN assay, generating results that compare well to gold standard microscopy for well-established positive (MMC and Etoposide) and negative (Mannitol) controls. The AAI software is based on a CNN that is designed to directly classify IFC imagery and does not rely on traditional feature-based image analysis methods which are rigid and require substantial optimization. Future inter-laboratory studies, using a comprehensive list of well-known chemicals across multiple cell lines, are required to develop fully validated models for genotoxicity and cytotoxicity scoring. Overall, this work establishes the feasibility of combining IFC and deep learning towards the development of a rapid and fully automated in vitro MN assay that may serve as a replacement for manual slide microscopy scoring, improving accuracy, reproducibility, and time-to-result for toxicity and biodosimetry applications.

## Methods

### Test chemicals

Test chemicals were purchased from MilliporeSigma (Billerica, MA, USA) and were selected since there is significant published literature that demonstrates their potential to induce MN across a wide range of doses^12,13,59–61^. MMC (CAS no. 50-07-7) is an alkylating agent that inhibits DNA synthesis by cross-linking the complementary strands of the DNA double helix. Etoposide (CAS no. 33419-42-0) inhibits DNA synthesis through the formation of a complex with topoisomerase II and DNA, which induces breaks and prevents repair by topoisomerase II binding. Mannitol sugar (CAS no. 69-65-8) is an osmotic diuretic that is metabolically inert in humans and was used as a negative control in this work as it has previously been shown to be MN negative. Mannitol and MMC were dissolved in sterile water while Etoposide was dissolved in DMSO.

### Cell line and culture conditions

Human lymphoblastoid TK6 cells were purchased from MilliporeSigma (cat. 95111735) and grown in HyClone RPMI-1640 media (SH30027.01; GE Healthcare Life Sciences, Utah, USA) supplemented with 10% fetal bovine serum (SH30071.03GE; Healthcare Life Sciences), 1% non-essential amino acid (13-114E; Lonza, NJ, USA), 1% sodium pyruvate (SH30239.01; GE Healthcare Life Sciences) and 1% penicillin-streptomycin (15070063; Gibco, Thermo Fisher Scientific, MA, USA). Cells were grown at 37 °C in a humidified atmosphere of 5% CO_2_ in air and were routinely passaged to ensure they remained in the exponential growth phase. Cells never exceeded passage 30 and their average doubling time was 14–15 h.

### Exposure and recovery schedules

Test chemicals were introduced into T25 culture flasks containing 10 mL of TK6 cells at a concentration of approximately 1–3 × 10^5^ cells/mL (non-Cyt-B assay) or 7–8 × 10^5^ cells/mL (Cyt-B assay). Mannitol and MMC were added at 10% (v/v) and Etoposide was added at 1% (v/v). Following a 3 h exposure time, cells were centrifuged to remove the test chemical and cultured in 10 mL of fresh media to recover for 24 h. For Cyt-B experiments, Cyt-B at a concentration of 3 μg/mL was added at the beginning of the recovery period. Solvent controls (sterile water or DMSO) were used as negative controls in each experiment. For MMC, all experiments were performed in duplicate and for Etoposide and Mannitol, all experiments were performed in triplicate.

### Culture harvesting, sample preparation, and staining

All culture harvesting, sample preparation, and cellular staining techniques used in this work have been described in detail in previous publications^26,27^. Briefly, for non-Cyt-B experiments, post-exposure cell counts to determine cytotoxicity were obtained using a TC-10 automated cell counter (Bio-Rad, Hercules, CA, USA). All samples were centrifuged and pellets were soft fixed in 75 mM KCl and 4% Formalin. Samples were then centrifuged, hard fixed in 4% Formalin and washed with wash buffer (1× PBS with 2% FBS). For microscope scoring, 5 µL from each sample was transferred to 30 µL of wash buffer and stained with Hoechst 33342 (H3570; Thermo Fisher Scientific, MA, USA). For IFC data acquisition, RNase (9001-99-4; MilliporeSigma) was added, samples were stained with Hoechst 33342 and incubated for 30 min at 37 °C. Following incubation, samples were centrifuged and the supernatant was pipetted off leaving approximately 30 μL.

### Microscope scoring

Slides were scored at ×100 magnification on a Nikon Eclipse E600 (Nikon, NY, USA) fluorescent microscope. For non-Cyt-B experiments, 1000 mononucleated (MONO) cells per culture were scored for the presence of MN. For Cyt-B experiments, 1000 binucleated (BN) cells were scored for the presence of MN to assess genotoxicity and an additional 500 cells per culture were scored and classified as either MONO, BN, or polynucleated (POLY) cells to assess cytotoxicity^4^. The POLY cells observed in this work had either three or four nuclei; cells with five or more nuclei were rarely observed. All cells were scored according to published scoring criteria by Fenech et al.^62^.

### IFC configuration and acquisition

All samples were run on an ImageStream^®X^ Mark II dual CCD camera system (Luminex Corporation, Seattle, WA, USA) at 40× magnification with the 405 nm laser set to 15 mW. Channels 1 and 9 were used to capture cytoplasmic images from the BF LED and Hoechst images (nuclei and MN) were captured in channel 7. For all samples, a data acquisition template^26^ was used to eliminate small and large debris, unfocused imagery and dimly stained events. All MMC samples were loaded manually and three data files of 15,000 events for the Cyt-B experiments per sample (45,000 events per culture) and three data files of 10,000 events for the non-Cyt-B experiments per sample (30,000 events per dose point) were collected. All Etoposide and Mannitol samples were acquired using the 96-well plate autosampler add-on which permits unattended data acquisition and eliminates the need for samples to be manually loaded. A total of 30,000 events were collected from each sample.

### IDEAS^®^ scoring

All IFC data was scored automatically for genotoxicity (MNed MONO in non-Cyt-B experiments and MNed BN cells in Cyt-B experiments) and cytotoxicity in Cyt-B experiments (MONO, BN and POLY cells) using data analysis templates in IDEAS^®^ that have been described in-depth in previous work^26^. Briefly, for the IDEAS^®^ scoring, a series of masks were created to highlight the main nuclei in MONO, BN and POLY cells and to highlight MN within MONO and BN cells. Features such as area, aspect ratio and circularity were applied to these masks to create a gating strategy that permitted images adhering to the published scoring criteria for the MN assay to be retained and scored^62^.

### Amnis^®^ AI (AAI) software description

The AAI software (v1.0) uses the Keras Application Programming Interface version 2.1.5^63^ with TensorFlow version 1.7.0 library^64^ to train deep learning models based on ground truth input data and to apply models to classify new data. The convolutional neural network (CNN) architecture used for training and classification is pre-configured based on the VGG16 network^65^ to work optimally on image data acquired on Amnis^®^ IFCs across a wide range of applications. Classification performance, usability and robustness were major considerations influencing the choice of the selected architecture. The user imports data to be used to build and train a model into the AAI software and all pixel values in each image are normalized to the range [0 1]. The ground truth populations for all critical event types are then populated by the user using the AI-assisted tagging tools (e.g. cluster and predict, Fig. 2) through a convenient user interface which eliminates the need for substantial computational expertise. Once the ground truth data has been populated, the AAI software splits the data into training, validation, and test sets using an 80/10/10 ratio–the validation and test datasets are never seen by the CNN during training. Class balancing and data augmentation are also performed to control classification bias and enhance the robustness of trained models. Once a model has been trained, it can then be easily applied to classify additional data. All computations were performed on an Intel® Xeon® E-2176M CPU @ 2.70 GHz machine with an NVIDIA^®^ Quadro^®^ P2000 GPU running Windows 10 Enterprise.

### Evaluation of genotoxicity and cytotoxicity

To evaluate genotoxicity and cytotoxicity, formulae in the OECD Test Guideline 487 for the in vitro MN assay were used^4^. For genotoxicity, the frequency of MN in BN cells and in MONO cells was quantified in Cyt-B and non-Cyt-B experiments, respectively. Also, in the IFC data, the frequency of MN in BN cells was quantified in non-Cyt-B experiments. For cytotoxicity, in non-Cyt-B experiments post-recovery cell counts were used and in Cyt-B experiments, the number of MONO, BN and POLY cells scored were used.

### Statistical analyses

The Fisher’s Exact Test (one-sided) was used to determine statistically significant increases in mean MN frequencies between solvent controls and dosed samples using the open source R software (version 4.0.2; https://www.r-project.org/) and the significance level was chosen to be 0.1% (α = 0.001). Error bars on microscopy data represent the standard deviation (SD) of the mean of the MN frequency from one slide per culture from triplicate cultures for Etoposide and Mannitol. For MMC, error bars on IDEAS^®^ and AAI data represent the SD of the mean of the MN frequency from six data file replicates (three data files per culture from duplicate cultures). For Etoposide and Mannitol, error bars on IDEAS^®^ and AAI data represent the SD deviation of the mean of the MN frequency from one data file per culture from triplicate cultures.

### Reporting summary

Further information on research design is available in the Nature Research Reporting Summary linked to this article.

## Supplementary information

Supplemental material

REPORTING SUMMARY

## Data Availability

The datasets generated during and/or analyzed during the current study are available from the corresponding author on reasonable request.

## Code availability

The code used in this study is not publically available as it is part of the Amnis^®^ AI software package that is currently patent pending. The Amnis^®^ AI software is available for purchase from Luminex Corporation.
