# Supplementary material for: The in vitro micronucleus assay using imaging flow cytometry and deep learning
Source: NPJ Syst Biol Appl. 2021 May 18;7:20. doi: 10.1038/s41540-021-00179-5 (PMC8131758; doi:10.1038/s41540-021-00179-5)
Supplement: Supplementary file 1 — Supplemental material [file 41540_2021_179_MOESM1_ESM.pdf]

Supplementary Table 1 | Parameters required to calculate cytotoxicity and genotoxicity for microscopy, IDEAS and AAI scoring in the Cyt-B assay. (**A-C**) Mitomycin C, (**D-F**) Etoposide and (**G-I**) Mannitol. For Mitomycin C, mean values are for duplicate cultures while for Etoposide and Mannitol, mean values are for triplicate cultures. Statistically significant increase in MN frequency are indicated with an asterisk (*P ≤ 0.001, Fishers Exact Test, one sided)

Supplementary Table 2 | Parameters required to calculate cytotoxicity and genotoxicity for microscopy, IDEAS and AAI scoring in the non-Cyt-B assay. (**A-C**) Mitomycin C, (**D-F**) Etoposide and (**G-I**) Mannitol. For Mitomycin C, mean values are for duplicate cultures while for Etoposide and Mannitol, mean values are for triplicate cultures. Statistically significant increase in MN frequency are indicated with an asterisk (*P ≤ 0.001, Fishers Exact Test, one sided)

Supplementary Table 1A – Mitomycin C, microscopy data and results

Dose (µg/mL)	Culture	BN cells without MN	BN cells with MN	Mean MNBN (%)	Fisher test value	MONO	POLY	Mean CBPI	Mean Toxicity (%)
0	1	986	14	1.30	---	141	135	2.01	---
	2	988	12			117	122		
0.125	1	963	37	3.95	<0.001	146	62	1.83	16.5
	2	958	42			158	76		
0.25	1	935	65	6.80*	<0.001	179	33	1.69	30.6
	2	929	71			203	42		
0.375	1	919	81	7.65*	<0.001	256	31	1.56	44.0
	2	928	72			243	27		
0.5	1	908	92	9.50*	<0.001	286	13	1.45	55.4
	2	902	98			306	25		

*statistically significant (P ≤ 0.001, Fishers Exact Test, two sided)

Supplementary Table 1B – Mitomycin C, IDEAS data and results

Dose (µg/mL)	Culture	Replicate	BN cells	BN cells with MN	Mean MNBN (SD) (%)	Fisher test value	MONO	POLY	Mean CBPI	Mean Toxicity (%)
0	1	1	3959	35	0.66 (0.13)	---	4250	869	1.69	---
		2	3570	22			4025	903		
		3	3741	20			3768	1032		
	2	1	4375	25			3370	1122		
		2	4426	27			3347	1121		
		3	4467	34			3497	1064		
0.125	1	1	4741	109	2.09 (0.269)*	<0.001	4738	285	1.42	38.2
		2	4563	113			4799	331		
		3	4517	87			4759	292		
	2	1	2678	52			7339	217		
		2	2689	47			7475	202		
		3	2652	57			7521	212		
0.25	1	1	4242	162	3.89 (0.419)*	<0.001	5391	145	1.42	38.5
		2	4215	191			5466	125		
		3	4178	176			5495	120		
	2	1	3768	129			6099	88		
		2	3678	141			6088	94		
		3	3553	125			6238	79		
0.375	1	1	2859	162	5.26 (0.341)*	<0.001	7975	67	1.30	56.6
		2	3003	142			7760	81		
		3	2974	152			7857	55		
	2	1	3232	166			7408	66		
		2	3290	183			7301	73		
		3	3247	173			7388	87		
0.5	1	1	2757	168	5.99 (0.717)*	<0.001	7739	79	1.23	66.4
		2	2675	142			7715	74		
		3	2616	180			7757	75		
	2	1	1800	115			8549	72		
		2	1846	116			8484	66		
		3	1874	93			8525	61		

*statistically significant (P ≤ 0.001, Fishers Exact Test, two sided)

Supplementary Table 1C – Mitomycin C, Amnis AI data and results

Dose (µg/mL)	Culture	Replicate	BN cells	BN cells with MN	Mean MNBN (SD) (%)	Fisher test value	MONO	POLY	Mean CBPI	Mean Toxicity (%)
0	1	1	4431	56	1.17 (0.109)	---	4931	1154	1.70	---
		2	4431	56			4931	1154		
		3	4115	48			4781	1213		
	2	1	4835	52			4109	1598		
		2	4920	57			4032	1598		
		3	4825	67			4199	1527		
0.125	1	1	4942	147	2.63 (0.221)*	<0.001	5403	293	1.40	41.7
		2	4746	125			5463	334		
		3	4710	120			5505	288		
	2	1	2857	69			8359	184		
		2	2909	70			8468	177		
		3	2874	80			8486	192		
0.25	1	1	4463	212	4.59 (0.380)*	<0.001	6271	150	1.40	42.3
		2	4454	232			6324	121		
		3	4374	206			6389	143		
	2	1	4047	174			6994	84		
		2	3891	171			7060	100		
		3	3815	159			7202	87		
0.375	1	1	3025	194	6.43 (0.268)*	<0.001	8599	78	1.30	58.0
		2	3140	189			8437	78		
		3	3103	198			8488	83		
	2	1	3399	227			8084	83		
		2	3482	227			7978	87		
		3	3438	207			8082	83		
0.5	1	1	2949	225	8.12 (1.17)*	<0.001	8325	93	1.23	67.3
		2	2834	174			8350	71		
		3	2764	237			8377	76		
	2	1	1949	179			9397	66		
		2	1992	167			9314	53		
		3	1954	131			9383	58		

*statistically significant (P ≤ 0.001, Fishers Exact Test, two sided)

Supplementary Table 1D – Etoposide, microscopy data and results

Dose (µg/mL)	Replicate	BN cells without MN	BN cells with MN	Mean MNBN (SD) (%)	Fisher test value	MONO	POLY	Mean CBPI	Mean Toxicity (%)
	1	990	10			75	151		
0	2	990	10	0.93 (0.12)	---	78	135	2.11	---
	3	991	8			98	137		
	1	968	32			91	114		
0.1	2	971	29	3.20 (0.30)	<0.001	123	29	1.88	21.3
	3	965	35			152	39		
	1	949	51			150	51		
0.15	2	955	45	4.70 (0.35)*	<0.001	81	61	1.87	21.8
	3	955	45			138	64		
	1	956	44			122	79		
0.2	2	948	52	5.13 (0.70)*	<0.001	126	84	1.88	21.1
	3	942	58			165	70		

*statistically significant (P ≤ 0.001, Fishers Exact Test, two sided)

Supplementary Table 1E – Etoposide, IDEAS data and results

Dose (µg/mL)	Replicate	BN cells	BN cells with MN	Mean MNBN (SD) (%)	Fisher test value	MONO	POLY	Mean CBPI	Mean Toxicity (%)
	1	7548	85			2794	1355		
0	2	8131	45	0.94 (0.33)	---	3454	1053	1.84	---
	3	7698	85			3295	1212		
	1	9326	368			5022	795		
0.125	2	8239	204	2.99 (0.83)*	<0.001	7344	115	1.60	28.8
	3	9400	239			8582	118		
	1	8822	382			7408	280		
0.25	2	9441	302	4.09 (0.80)*	<0.001	8140	169	1.57	31.9
	3	9659	459			7738	485		
	1	9057	583			7327	486		
0.375	2	7949	469	5.87 (0.59)*	<0.001	4597	342	1.60	28.4
	3	9379	494			8799	277		

*statistically significant (P ≤ 0.001, Fishers Exact Test, two sided)

Supplementary Table 1F – Etoposide, Amnis AI data and results

Dose (µg/mL)	Replicate	BN cells	BN cells with MN	Mean MNBN (SD) (%)	Fisher test value	MONO	POLY	Mean CBPI	Mean Toxicity (%)
	1	8047	163			3603	6619		
0	2	8836	117	1.69 (0.35)	---	4354	5298	2.10	---
	3	8300	149			4082	5846		
	1	9456	558			5961	3645		
0.125	2	8376	341	4.49 (0.94)*	<0.001	8478	697	1.67	39.3
	3	9567	396			9830	798		
	1	8948	604			8731	1420		
0.25	2	9670	537	5.95 (0.59)*	<0.001	9258	1096	1.64	41.7
	3	9698	647			8656	2228		
	1	8900	872			8304	2271		
0.375	2	7845	687	7.98 (0.99)*	<0.001	7774	1762	1.66	40.0
	3	9181	686			9753	1574		

*statistically significant (P ≤ 0.001, Fishers Exact Test, two sided)

Supplementary Table 1G – Mannitol, microscopy data and results

Dose (mg/mL)	Replicate	BN cells without MN	BN cells with MN	Mean MNBN (SD) (%)	Fisher test value	MONO	POLY	Mean CBPI	Mean Toxicity (%)
	1	995	5			89	130		
0	2	995	5	0.43 (0.12)	---	70	135	2.11	---
	3	997	3			92	148		
	1	989	11			88	123		
1	2	992	8	0.80 (0.30)	0.386	63	144	2.11	-0.5
	3	995	5			82	137		
	1	994	6			64	125		
2	2	993	7	0.63 (0.06)	0.753	84	136	2.12	-1.0
	3	994	6			81	147		
	1	993	7			56	170		
3	2	987	13	1.03 (0.31)	0.178	91	132	2.15	-3.5
	3	989	11			83	149		

Supplementary Table 1H – Mannitol, IDEAS data and results

Dose (mg/mL)	Replicate	BN cells	BN cells with MN	Mean MNBN (SD) (%)	Fisher test value	MONO	POLY	Mean CBPI	Mean Toxicity (%)
	1	8879	66			3583	3512		
0	2	9317	70	0.76 (0.02)	---	3903	2936	1.96	---
	3	9107	71			4170	3250		
	1	9869	67			3752	3461		
1	2	9272	59	0.68 (0.04)	0.543	4827	2549	1.94	2.2
	3	9339	67			3847	3355		
	1	9650	53			3764	3597		
2	2	9775	73	0.71 (0.15)	0.798	4357	2833	1.95	0.8
	3	9447	80			3972	3255		
	1	9960	58			3857	3131		
3	2	9943	69	0.65 (0.06)	0.386	3743	3277	1.96	-0.3
	3	10280	68			3888	3169		

Supplementary Table 1I – Mannitol, Amnis AI data and results

Dose (µg/mL)	Replicate	BN cells	BN cells with MN	Mean MNBN (SD) (%)	Fisher test value	MONO	POLY	Mean CBPI	Mean Toxicity (%)
	1	9392	150			4704	5293		
0	2	9939	145	1.45 (0.12)	---	5066	4612	2.00	---
	3	9676	131			5173	4837		
	1	10444	122			4709	5067		
1	2	9874	135	1.26 (0.10)	0.271	5912	3912	1.97	2.2
	3	9946	130			4874	5037		
	1	10221	127			4877	5210		
2	2	10494	136	1.30 (0.09)	0.397	5548	4126	1.98	1.6
	3	10028	142			5047	4988		
	1	10499	117			4794	4726		
3	2	10553	152	1.24 (0.16)	0.203	4755	4968	2.00	-0.6
	3	10887	132			4828	4842		

Supplementary Table 2A – Mitomycin C, microscopy data and results (no Cyt-B)

Dose (µg/mL)	Culture	Pre-exposure cell counts (x10^5 cells/mL)	Pre-exposure cell counts (x10^5 cells/mL)	Mean Toxicity (%)	Mono cells without MN	Mono cells with MN	Mean MN Mono (%)	Fisher test value
0	1	3.25	9.96	---	989	11	1.00	---
	2		10.97		991	9		
0.125	1	3.25	8.53	23.5	976	24	2.55*	0.0157
	2		7.38		973	27		
0.25	1	3.25	6.60	39.9	950	50	4.75*	<0.001
	2		6.53		955	45		
0.375	1	3.25	5.20	54.2	932	68	6.65*	<0.001
	2		5.90		935	65		
0.5	1	3.25	4.21	76.0	921	79	7.70*	<0.001
	2		4.40		925	75		

*statistically significant (P < 0.001, Fishers Exact Test, two sided)

Supplementary Table 2B – Mitomycin C, IDEAS data and results (no Cyt-B)

Dose (µg/mL)	Culture	Replicate	Mono cells	Mono cells with MN	Mean MN Mono (SD) (%)	Fisher test value	BN cells	BN cells with MN	% BN cells	Mean MNBN (SD) (%)
0	1	1	8977	49	0.55 (0.075)	---	67	3	0.67	3.77 (1.61)
		2	8942	59			83	2	0.83	
		3	8957	51			79	2	0.79	
	2	1	8973	38			77	2	0.77	
		2	8825	44			92	6	0.92	
		3	8828	49			73	3	0.73	
0.125	1	1	8209	230	2.67 (0.269)*	<0.001	169	18	1.69	12.2 (2.51)
		2	8161	215			179	29	1.79	
		3	6766	193			140	14	1.40	
	2	1	8284	209			189	18	1.89	
		2	8407	218			151	16	1.51	
		3	8438	181			168	21	1.68	
0.25	1	1	7139	342	4.65 (0.174)*	<0.001	275	40	2.75	12.8 (1.16)
		2	7116	327			278	31	2.78	
		3	7090	313			318	40	3.18	
	2	1	6662	294			306	30	3.06	
		2	7199	328			352	40	3.52	
		3	7170	307			348	44	3.48	
0.375	1	1	6359	325	5.08 (0.299)*	<0.001	443	64	4.43	16.5 (1.35)
		2	6501	311			463	74	4.63	
		3	6540	330			500	84	5.00	
	2	1	6381	291			336	46	3.36	
		2	6370	296			335	53	3.35	
		3	6460	323			338	60	3.38	
0.5	1	1	5636	298	6.15 (0.616)*	<0.001	350	60	3.50	16.8 (1.63)
		2	5710	309			283	47	2.83	
		3	5561	315			337	49	3.37	
	2	1	6184	412			375	55	3.75	
		2	6104	369			352	54	3.52	
		3	6164	401			340	61	3.40	

*statistically significant (P < 0.001, Fishers Exact Test, two sided)

Supplementary Table 2C – Mitomycin C, Amnis AI data and results (no Cyt-B)

Dose (µg/mL)	Culture	Replicate	Mono cells	Mono cells with MN	Mean MN Mono (SD) (%)	Fisher test value	BN cells	BN cells with MN	%BN cells	Mean MNBN (SD) (%)
0	1	1	9363	63	0.60 (0.054)	---	86	5	0.81	5.81 (0.887)
		2	9305	63			96	4	0.92	
		3	9317	58			96	6	0.90	
	2	1	9321	55			85	5	0.80	
		2	9273	52			101	6	0.95	
		3	9282	51			88	6	0.82	
0.125	1	1	8494	273	3.01 (0.303)*	<0.001	235	64	1.71	25.9 (1.55)
		2	8480	264			243	61	1.82	
		3	7454	288			188	49	1.39	
	2	1	8601	261			261	61	2.00	
		2	8674	270			187	49	1.38	
		3	8736	250			242	67	1.75	
0.25	1	1	7634	465	5.35 (0.233)*	<0.001	425	133	2.92	28.5 (1.95)
		2	7631	481			395	114	2.81	
		3	7610	473			433	128	3.05	
	2	1	7182	436			436	114	3.22	
		2	7653	428			469	124	3.45	
		3	7564	429			479	139	3.40	
0.375	1	1	6955	471	6.02 (0.169)*	<0.001	616	198	4.18	30.5 (1.15)
		2	7056	501			658	196	4.62	
		3	7162	468			695	221	4.74	
	2	1	6951	465			458	140	3.18	
		2	6961	490			423	124	2.99	
		3	7027	488			445	133	3.12	
0.5	1	1	6387	526	7.89 (0.594)*	<0.001	428	147	2.81	32.6 (3.09)
		2	6419	568			391	140	2.51	
		3	6289	580			397	114	2.83	
	2	1	6543	675			507	146	3.61	
		2	6557	609			490	170	3.20	
		3	6566	682			471	157	3.14	

*statistically significant (P < 0.001, Fishers Exact Test, two sided)

Supplementary Table 2D – Etoposide, microscopy data and results (no Cyt-B)

Dose (µg/mL)	Replicate	Pre-exposure cell counts (x10^5 cells/mL)	Pre-exposure cell counts (x10^5 cells/mL)	Mean Toxicity (%)	Mono cells without MN	Mono cells with MN	Mean MN Mono (SD) (%)	Fisher test value
	1		8.65		996	4		
0	2	1.98	9.17	---	991	9	0.67 (0.252)	---
	3		10.25		993	7		
	1		5.73		969	31		
0.1	2	1.98	6.59	31.2	971	29	2.80 (0.361)*	<0.001
	3		4.98		976	24		
	1		5.18		961	39		
0.15	2	1.98	4.90	37.9	954	46	4.13 (0.404)*	<0.001
	3		5.50		961	39		
	1		3.82		946	54		
0.2	2	1.98	5.02	47.5	945	55	5.13 (0.551)*	<0.001
	3		4.58		955	45		

*statistically significant (P < 0.001, Fishers Exact Test, two sided)

Supplementary Table 2E – Etoposide, IDEAS data and results (no Cyt-B)

Dose (µg/mL)	Replicate	Mono cells	Mono cells with MN	Mean MN Mono (SD) (%)	Fisher test value	BN cells	BN cells with MN	%BN cells	Mean MNBN (SD) (%)
	1	27322	139			149	4	0.54	
0	2	27546	125	0.49 (0.0282)	---	116	8	0.42	6.12 (3.12)
	3	27615	135			91	8	0.33	
	1	23971	572			327	42	1.35	
0.1	2	26043	576	2.37 (0.100)*	<0.001	409	70	1.55	16.5 (2.97)
	3	25924	619			458	82	1.74	
	1	25449	744			530	87	2.04	
0.15	2	25203	795	3.09 (0.131)*	<0.001	540	85	2.10	17.7 (0.421)
	3	25228	741			587	97	2.27	
	1	23670	872			751	111	3.08	
0.2	2	24619	834	3.69 (0.185)*	<0.001	640	125	2.53	17.3 (2.63)
	3	24021	878			887	127	3.56	

*statistically significant (P < 0.001, Fishers Exact Test, two sided)

Supplementary Table 2F – Etoposide, Amnis AI data and results (no Cyt-B)

Dose (µg/mL)	Replicate	Mono cells	Mono cells with MN	Mean MN Mono (SD) (%)	Fisher test value	BN cells	BN cells with MN	%BN cells	Mean MNBN (SD) (%)
	1	28754	149			191	13	0.66	
0	2	28905	136	0.50 (0.028)	---	156	20	0.54	12.4 (5.41)
	3	28979	151			108	19	0.37	
	1	27024	773			454	148	1.70	
0.1	2	27836	766	2.77 (0.091)*	<0.001	574	195	2.08	32.9 (0.966)
	3	27650	814			626	201	2.28	
	1	27321	1040			697	252	2.58	
0.15	2	27172	1090	3.70 (0.138)*	<0.001	777	277	2.89	34.9 (1.63)
	3	27139	1010			788	261	2.93	
	1	26053	1240			947	298	3.68	
0.2	2	26838	1152	4.33 (0.214)*	<0.001	877	321	3.30	34.2 (2.58)
	3	26201	1184			1197	414	4.57	

*statistically significant (P < 0.001, Fishers Exact Test, two sided)

Supplementary Table 2G – Mannitol, microscopy data and results (no Cyt-B)

Dose (mg/mL)	Replicate	Pre-exposure cell counts (x10^5 cells/mL)	Pre-exposure cell counts (x10^5 cells/mL)	Mean Toxicity (%)	Mono cells without MN	Mono cells with MN	Mean MN Mono (SD) (%)	Fisher test value
	1		12.10		992	8		
0	2	2.80	11.90	---	996	4	0.50 (0.26)	---
	3		11.80		997	3		
	1		11.70		996	4		
1	2	2.80	10.20	2.2	996	4	0.43 (0.06)	1.0
	3		12.80		995	5		
	1		10.90		996	4		
2	2	2.80	12.90	0.2	996	4	0.47 (0.12)	1.0
	3		11.90		994	6		
	1		11.80		991	9		
3	2	2.80	12.00	1.4	994	6	0.63 (0.25)	0.774
	3		11.30		996	4		

Supplementary Table 2H – Mannitol, IDEAS data and results (no Cyt-B)

Dose (mg/mL)	Replicate	Mono cells	Mono cells with MN	Mean MN Mono (SD) (%)	Fisher test value	BN cells	BN cells with MN	%BN cells	Mean MNBN (SD) (%)
	1	27933	110			111	4	0.40	
0	2	27860	124	0.40 (0.046)	---	131	7	0.47	3.82 (1.42)
	3	28006	99			119	3	0.42	
	1	27812	123			167	5	0.60	
1	2	28114	111	0.39 (0.052)	0.892	137	2	0.48	2.65 (1.06)
	3	27950	94			86	3	0.31	
	1	27665	109			148	5	0.53	
2	2	27999	100	0.36 (0.044)	0.488	150	5	0.53	3.17 (0.323)
	3	28159	93			143	4	0.51	
	1	27974	113			102	4	0.36	
3	2	28133	103	0.37 (0.053)	0.682	100	6	0.35	5.30 (1.20)
	3	27978	97			117	7	0.42	

Supplementary Table 2I – Mannitol, Amnis AI data and results (no Cyt-B)

Dose (mg/mL)	Replicate	Mono cells	Mono cells with MN	Mean MN Mono (SD) (%)	Fisher test value	BN cells	BN cells with MN	%BN cells	Mean MNBN (SD) (%)
	1	28932	125			122	24	0.50	
0	2	29068	106	0.38 (0.040)	---	104	18	0.42	12.7 (5.07)
	3	29007	104			121	9	0.45	
	1	28987	109			157	13	0.58	
1	2	28889	126	0.40 (0.009)	0.791	168	11	0.62	8.01 (2.06)
	3	28841	113			158	18	0.61	
	1	28916	114			105	9	0.39	
2	2	28928	115	0.42 (0.040)	0.473	149	15	0.56	7.50 (1.86)
	3	28801	139			190	11	0.69	
	1	28899	124			127	15	0.49	
3	2	28816	141	0.46 (0.031)	0.143	144	15	0.55	10.1 (0.586)
	3	28879	139			131	15	0.50	
